# Vocal and motor behaviors as a possible expression of gastrointestinal problems in preschoolers with Autism Spectrum Disorder

**DOI:** 10.1186/s12887-019-1841-8

**Published:** 2019-11-29

**Authors:** Margherita Prosperi, Elisa Santocchi, Filippo Muratori, Chiara Narducci, Sara Calderoni, Raffaella Tancredi, Maria Aurora Morales, Letizia Guiducci

**Affiliations:** 1 Department of Developmental Neuroscience, IRCCS Fondazione Stella Maris, Viale del Tirreno 331, 56128 Calambrone, Pisa, Italy; 2 Department of Clinical and Experimental Medicine, University of Pisa, Pisa, Italy; 3 Child and Adolescent Neuropsychiatry Unit Department of Biomedical Science, University of Cagliari and “Antonio Cao” Paediatric Hospital, “G. Brotzu” Hospital trust, Cagliari, Italy; 4 Institute of Clinical Physiology, CNR, National Research Council, Pisa, Italy

**Keywords:** Abdominal pain, Diarrhea, Constipation, Gastroesophageal reflux, Sleep problems

## Abstract

**Background:**

Gastrointestinal (GI) problems are one of the most frequent comorbidities in Autism Spectrum Disorder (ASD) but can be under-recognized due to the concomitant communication difficulties of this population. Accordingly, some associated behaviors (AB) such as verbal and motor behaviors (VB and MB, respectively) have been identified as a possible expression of an underlying GI problem and evaluated through an ad hoc questionnaire (the Associated Behaviors Questionnaire -ABQ-). The aims of this study were to investigate the presence and the type of AB in an Italian sample of ASD preschoolers, and to determine their correlations with GI problems.

**Methods:**

We included 85 ASD preschoolers (mean age 4.14 years; SD 1.08) splitted into two groups (GI and No-GI) through the GI Severity Index instrument. AB were evaluated through the ABQ that includes VB, MB and Changes in overall state (C) clusters. Specific tools were administered to evaluate the ASD core ad associated symptoms, as well as the intellective and adaptive functioning.

**Results:**

The GI group (*N* = 30) showed significantly higher scores in all the three ABQ areas (VB, MB and C) than the No-GI group (*N* = 55), with a positive correlation between GI symptoms and some specific AB as well as ABQ Total score. By dividing the whole sample in verbal and non-verbal individuals, both specific and shared AB emerged in the two groups.

**Conclusions:**

Our results alert clinicians to consider behavioral manifestations as a possible expression of GI problems in ASD subjects. Therefore, the evaluation of AB may be useful to identify the presence of GI problems in the ASD populations, and especially in non-verbal ASD children.

## Background

Autism spectrum disorders (ASD) are a group of neurodevelopmental disorders characterized by social and communication impairment along with the presence of repetitive and restrictive behaviors [1]. According to a recent Italian population study, the prevalence of ASD in children aged 7–9 years is of about one in 87 [2].

A number of co-occurring health conditions were described in ASD (for a recent systematic review see Muskens et al. [3]), contributing to the considerable ASD clinical variability, and impacting on quality of life both of the children and their parents [4]. Within the medical comorbidities, gastrointestinal (GI) problems are widely reported by several studies [5–15], and among them constipation and diarrhea were the most commonly observed symptoms. Marked variation exists *among studies* on the *prevalence* of GI problems in ASD pediatric population, partly related to the different tools used for the GI assessment [15, 16]. Specifically, the prevalence is greater in investigations that include functional and idiopathic GI symptoms, and examine data from parents’ reports considering longer time intervals [17, 18] than in studies reporting organic GI diseases, clinician’s evaluation, or considering shorter time ranges. Moreover, Gorrindo et al. [19] reported that the majority of GI symptoms in children with ASD are due to functional causes.

More anxiety and sensory over-responsiveness [20], irritability [8], sleep problems [21], rigid-compulsive behaviors [13, 22], opposition and emotional dysregulation, as well as anxiety and affective disorders [23] were described in ASD subjects with GI impairment in comparison with ASD children without GI symptoms. Currently, there is a paucity of literature on differences in clinical manifestations between subjects with functional GI problems and subjects with organic GI problems [15].

Crucially, autism-related communication impairments may lead to unusual manifestations of GI disorders [24, 25]. In fact, many children with ASD have limited expressive language abilities as well as sensory processing impairments that could prevent the effective communication and/or the proper localization of pain of some GI symptoms [24, 26, 27]. Therefore, in ASD children clinical tools based on observable manifestations could be more useful than those based on reported symptoms.

Accordingly, in the ‘Consensus Report on the evaluation, diagnosis and treatment of GI disorders in ASD subjects’ [24] several behaviors (Verbal Behaviours “VB”, Motor Behaviours “MB” and Changes in Overall State “C”) that could be considered as alternative ways of expressing GI disorders in individuals unable to effectively communicate their discomfort are reported. The authors called all these behaviors “Associated Behaviors” (AB) [25].

Two studies [28, 29] tested AB in ASD children in order to determine if they occurred more frequently when a GI disorder was present. The first investigation evaluated 487 ASD children with and without GI problems and showed that unusual sleeping or eating habits as well as oppositional behaviors were significantly associated with GI problems [28]. In a subsequent study [29], the authors proposed a brief parent-report screen able to identify functional constipation, functional diarrhea, and gastroesophageal reflux disease (GERD) in ASD children, which minimally relied upon the child’s ability to report or to localize pain. This screening tool asked about the recurring motor acts described in the Consensus report [24] proving to be particularly useful for identifying cases of GERD.

The exploration of the association between GI symptoms and AB could allow to better identify the presence of GI problems, improving care and quality of life of ASD subjects.

Therefore, the main aims of our study are:

1. To investigate the correlation between GI symptoms and the presence and the type of the AB reported in the Consensus report [24] in an Italian sample of preschoolers with ASD;
2. To evaluate possible differences in the expression of AB between verbal and non-verbal ASD subjects with GI problems.

## Methods

### Participants

A total of 85 preschoolers who received a diagnosis of ASD according to the Diagnostic and Statistical Manual of Mental Disorders-5th Edition (DSM-5) [1] criteria were included in this observational case-control study, comprising 71 males (83.5%) and 14 females (16.5%), (mean age [SD] = 4.14 [1.08] years; range 2.18–6.11 years). Children were recruited from November 2015 to February 2018 at the ASD Unit of the IRCCS Stella Maris Foundation (Pisa, Italy), a tertiary care university hospital.

ASD patients performed the recommended clinical tests to rule-out medical causes of ASD, including neurological examination, audiometry, evaluation of thyroid function, array comparative genomic hybridization, DNA analysis of FRA-X and screening tests for inborn errors of metabolism. The subjects with a diagnosis of organic GI Disorder (i.e. GERD, food allergies, IBD, coeliac disease) were excluded because they are less frequently referred to our ASD Unit than subjects with functional GI symptoms, possibly causing a numerical imbalance. Subjects with special diet (i.e. gluten-free diet, casein-free diet, high-protein diet, ketogenic diet) were also excluded.

The study protocol was approved by the Pediatric Ethic Committee of the Tuscany Region (Protocol Number: 126/2014), with written informed consent obtained from a parent/guardian of each participant. The study was conducted in accordance with the 1964 Declaration of Helsinki and its later amendments, and the International Conference on Harmonization Guidelines for Good Clinical Practice.

### Instruments

The *Gastrointestinal Severity Index (GSI)* [30] is an instrument designed to identify signs and symptoms of GI distress commonly reported by parents of children with ASD. The GSI includes nine variables: the first six exploring specific GI symptoms (constipation, diarrhea, stool consistency, stool smell, flatulence, abdominal pain) and the last three exploring unexplained daytime irritability, night-time awakening, and abdominal tenderness. Data about the first 8 variables were filled out by parents and rated on a three-point Likert scale (0–2) based on the severity of symptoms occurring in the last 3 months. Abdominal tenderness was evaluated by a physician through the abdominal palpation.

The *Associated Behaviors Questionnaire (ABQ)* is a questionnaire completed by parents about the behaviors reported by Buie and colleagues [24] as probably associated to pain or abdominal discomfort in ASD subjects. It includes 22 items, rated on a three-point Likert scale (absent behavior, not very present/infrequent behavior, very pronounced/very frequent behavior), which can be grouped into three clusters [24] (see Table 1).

**Table 1:** Behaviors assessed by ABQ as possible markers of abdominal discomfort in individuals with ASD

VB	MB	C
Frequent clearing of throat	Facial grimacing	Sleep disturbances (difficulty getting to sleep, difficulty staying asleep)
Swallowing and/or tics	Gritting teeth	Increased irritability (exaggerated responses to stimuli)
Screaming	Wincing	Non-compliance with demands that typically elicit an appropriate response
Sobbing for no reason at all	Constant eating/drinking/swallowing	
Sighing and/or whining	Mouthing behaviors as chewing on clothes, pica	
Moaning and/or groaning	Application of pressure to abdomen	
Delayed echolalia that includes reference to pain or stomach and direct verbalizations about it	Tapping behaviors	
	Any unusual posturing	
	Agitation as jumping up and down	
	Unexplained increase in repetitive behaviors	
	Self-injurious behaviors	
	Aggressive behaviors: onset of or increase	

Abbreviations: *VB* Verbal behaviors, *MB* Motor behaviors, *C* Changes in overall state

The *Autism Diagnostic Observation Schedule-Second Edition (ADOS-2)* [31, 32] is a semi-structured assessment of communication, social interactions, play, imagination, and stereotyped or repetitive behaviors used as the golden standard tool for the diagnosis of ASD [33]. It includes five modules (Toddler, 1, 2, 3, 4), each tailored to children language level and age. We used Toddler Module for 3 children, Module 1 for 71 children, and Module 2 for 11 children. The overall score is obtained from two separate domains, Social Affect and Restricted, Repetitive Behaviors, and from the sum of both. Raw scores were converted into calibrated scores through the Calibrated Severity Score (ADOS-CSS) [34–36]. The A1 score of the ADOS-2 (“Total level of spoken language non-echolalic”) was used to differentiate non-verbal (those with absent language or less than 5 words) from verbal children.

The *Repetitive Behavior Scale-Revised (RBS-R)* [37] is a questionnaire completed by parents about the presence of a broad spectrum of repetitive behaviors. In our study, we used the Italian version [38]. Total Score (the sum of all Subscales-Scores), Total Number-Endorsed Score (the sum of all Number-Endorsed–Subscale-Scores), and Global Rating Score (the judgment of the parents of the RRB interference in the child’s life summarized in a score that ranges from 1 to 100) were calculated.

The *Griffiths Mental Development Scales-Extended Revised (GMDS-ER)* [39, 40] are a developmental assessment procedure including five different subscales (Locomotor, Personal-Social, Hearing and Speech, Eye and Hand Coordination and Performance) used to assess intellectual abilities. The subscales yield standardized scores for each domain. We have used the Performance subscale to measure the non-verbal skills of each child; 65 out of 85 children were evaluable with this test.

The *Vineland Adaptive Behavior Scales-Second edition (VABS-II)* [41] is a parent interview that assesses adaptive functioning in the areas of communication, daily living, socialization, and motor skills. Scores obtained in these areas flow into an adaptive behavior composite score.

The *Child Behavior CheckList 1.5–5 (CBCL 1.5–5)* [42, 43] is a parent-report questionnaire that includes 100 statements about child’s behaviors. These items provide score for seven syndrome scales: Emotionally Reactive, Anxious/Depressed, Somatic Complaints, Withdrawn, Aggressive Behavior, Attention Problems and Sleep Problems. Scores of syndrome scales flow into three summary scales (i.e., Internalizing, Externalizing and Total Problems). Finally, it provides scores for five DSM-Oriented scales: Affective Problems, Anxiety Problems, Pervasive Developmental Disorder (PDD) Problems, Attention Deficit Hyperactivity Disorder (ADHD) Problems, and Oppositional Defiant Problems. Demographic and clinical characteristics of participants are reported in Table 2.

**Table 2:** Clinical characteristics of the whole sample and statistical comparisons of clinical variables between GI and No-GI groups

Clinical variables	Values	GI	No-GI	*p* (No-GI vs GI)
Age (years) mean ± SD	4.14 ± 1.08	4.06 (1.05)	4.19 (1.10)	NS
Males *n* (%)	71 (83.5%)	26 (36.6%)	45 (63.4%)	NS
Females *n* (%)	14 (16.5%)	4 (28.6%)	10 (71.4%)	NS
ADOS-2				
CSS Social Affect (mean ± SD)	6.43 ± 2.05	6.77 (1.91)	6.29 (2.05)	NS
CSS Restricted Repetitive Behaviors (mean ± SD)	8.23 ± 1.46	8.43 (1.48)	8.20 (1.45)	NS
CSS Total (mean ± SD)	7.05 ± 1.85	7.43 (2.01)	6.95 (1.68)	NS
GMDS-ER (65 out of 85 children were evaluable with this test)				
Locomotor (mean ± SD)	71.85 ± 18.53	70.92 (16.19)	72.88 (20.03)	NS
Personal-Social (mean ± SD)	54.27 ± 20.00	48.96 (17.48)	57.55 (20.72)	NS
Hearing and Speech (mean ± SD)	46.03 ± 22.38	42.87 (20.20)	47.07 (23.32)	NS
Eye-Hand Coordination (mean ± SD)	66.64 ± 22.13	62.32 (20.07)	67.72 (24.34)	NS
Performance (mean ± SD)	70.75 ± 23.33	67.80 (21.62)	72.26 (24.36)	NS
VABS-II				
Communication (mean ± SD)	50.86 ± 17.79	45.47 (15.22)	54.46 (18.80)	0.0274
Daily Living (mean ± SD)	66.56 ± 17.07	61.13 (14.29)	69.07 (17.51)	0.0365
Socialization (mean ± SD)	63.55 ± 15.02	60.93 (13.59)	65.18 (15.83)	NS
Motor Skills (mean ± SD)	71.88 ± 17.85	69.83 (16.34)	74.71 (17.69)	NS
Composite Score (mean ± SD)	59.40 ± 19.53	54.40 (18.51)	62.29 (20.03)	NS
CBCL 1.5–5				
Internalizing Problems (mean ± SD)	63.85 ± 9.06	67.48 (7.80)	62.06 (9.04)	0.0065
Externalizing Problems (mean ± SD)	57.10 ± 9.09	59.07 (7.55)	55.82 (9.61)	NS
Total Problems (mean ± SD)	62.28 ± 10.51	65.35 (10.02)	60.62 (10.30)	0.0469
RBS-R				
Total Score (mean ± SD)	19.87 ± 13.87	20.07 (13.27)	19.76 (14.29)	NS
Total Endorsed Score (mean ± SD)	12.76 ± 7.27	13.03 (6.78)	12.62 (7.57)	NS
Global Score (mean ± SD)	44.18 ± 27.24	60.24 (20.77)	38.12 (27.06)	0.0016

Abbreviations: *SD* Standard deviation, *ADOS-2* Autism Diagnostic Observation Schedule-2, *CSS* Calibrated Severity Score, *GMDS-ER* Griffiths Mental Development Scales-Extended Revised, *VABS-II* Vineland Adaptive Behavior Scales-II, *CBCL 1.5–5* Child Behavior Checklist 1.5–5, *RBS-R* Repetitive Behaviors Scale Revised

Information about pharmacological treatments and food supplements in the previous 3 months were collected: parents reported an acute or episodic administration of antibiotics (28.2%), probiotics (8.2%), NSAIDs or paracetamol (14.1%), steroids (8.2%), other drugs without effects on GI symptoms (36.5%), and a chronic administration of osmotic laxatives (12.9%).

### Statistical analysis

The Analysis of Variance (ANOVA) was used to compare males vs females for all variables, GI vs No-GI subjects and verbal vs non-verbal subjects for the scores at the ABQ.

Correlations and regression analysis were computed to study the relationship between the scores on GI Severity Index and the scores reported on the ABQ. The multivariate regression was used to investigate the impact of the clinical variables on the differences between the GI and the No-GI groups.

All statistical analyses were performed using the software Statistical Package for Social Sciences (SPSS), version 17.0 for Windows [44], with *p* = 0.05 as the significance level.

### Procedure

The correlations of the GI symptoms with the ABQ Total Score, with the three summary clusters (VB, MB and C) and with each single item of the ABQ were examined considering only the first 6 items of GSI (i.e. the 6-GI score) to avoid a possible bias due to the overlapping of survey areas between the other GSI items and ABQ items.

A total score of 4 or above on GSI (with at least 3 score points from the first six items) was considered clinically significant for the classification of a subject within the GI group.

GI vs No-GI subjects and verbal vs non-verbal subjects were compared, respectively, for their mean scores at the ABQ considering the ABQ Total Score, the three summary clusters (VB, MB and C) and each single item of the ABQ.

The correlation of the 6-GI score with the three summary clusters and with the single items of the ABQ was examined in verbal vs non-verbal subjects.

The scores on ADOS-2, VABS-II, RBS-R, CBCL and GMDS-ER have been used to better characterize our sample and they have been compared between the GI and the No-GI groups.

## Results

No statistically significant differences were found comparing males vs females for all variables, therefore the sample was considered as a unit. Thirty children (35%) were in the GI group, and 55 (65%) in the No-GI group. There were no significant differences in clinical variables between the GI and the No-GI groups, with the exception of the Global Score of the RBS-R, the Internalizing and Externalizing problem scores of the CBCL (all significantly higher in the GI group than in the No-GI group), and of the Communication and Daily Living adaptive scores of the VABS-II (significantly higher in the No-GI group than in the GI group) (see Table 2).

Therefore, we examined the correlations of the 6-GI scores with the ABQ Total Scores in the whole sample (R = 0.422, B = 1.394, *p* = < 0.001). In the multiple regression model, the correlation between the 6-GI scores and the ABQ Total Scores persisted even after correction for the variables that significantly differ between the GI and the No-GI group (*p* = 0.0026).

As far as the single AB is concerned, a positive correlation between the 6-GI scores and VB total score (*p* = 0.009), and with “frequent clearing of throat, swallowing and/or tics” (*p* = 0.043), “screaming” (*p* = 0.048), “sighing and/or whining” (*p* = 0.039), “moaning and/or groaning” (*p* = 0.003), and “direct verbalization about pain or stomach” (*p* = 0.015) scores was detected.

In addition, a positive correlation between the 6-GI scores and MB total score (*p* = 0.015), “facial grimacing” (*p* = 0.010), “constant eating/drinking/swallowing” (*p* = 0.002), “application of pressure to abdomen” (*p* = 0.032), and “aggressive behaviors” (*p* = 0.032) scores was observed.

Moreover, a positive correlation between the 6-GI scores and C total score (*p* = 0.041) was found.

The statistically significant correlations of the 6-GI scores with the three summary clusters (VB, MB and C) and with the singles items of the ABQ are reported in Table 3.

**Table 3:** Correlations between the 6-GI and the ABQ scores. Only statistically significant (*p* < 0.05) scores are reported

ABQ clusters and items	Correlations ABQ/6-GI scores
Whole sample	
VB (overall score)	*R* = 0.281 B = 0.294 *p* = 0.009
Frequent clearing of throat, swallowing and/or tics	*R* = 0.258 B = 0.075 *p* = 0.043
Screaming	*R* = 0.252 B = 0.110 *p* = 0.048
Sighing and/or whining	*R* = 0.263 B = 0.089 *p* = 0.039
Moaning and/or groaning	*R* = 0.375 B = 0.128 *p* = 0.003
Direct verbalization about pain or stomach	*R* = 0.309 B = 0.084 *p* = 0.015
MB (overall score)	*R* = 0.264 B = 0.526 *p* = 0.015
Facial grimacing	*R* = 0.325 B = 0.100 *p* = 0.010
Constant eating/drinking/swallowing	*R* = 0.387 B = 0.126 *p* = 0.002
Application of pressure to abdomen	*R* = 0.273 B = 0.091 *p* = 0.032
Aggressive behaviors	*R* = 0.273 B = 0.091 *p* = 0.032
C (overall score)	*R* = 0.222 B = 0.181 *p* = 0.041
Verbal children	
VB (overall score)	–
Frequent clearing of throat, swallowing and/or tics	*R* = 0.411 B = 0.119 *p* = 0.022
Direct verbalization about pain or stomach	*R* = 0.423 B = 0.135 *p* = 0.018
MB (overall score)	–
Constant eating/drinking/swallowing	*R* = 0.474 B = 0.184 *p* = 0.007
C (overall score)	–
Sleep disturbances	*R* = 0.437 B = 0.207 *p* = 0.014
Non-verbal children	
VB (overall score)	*R* = 0.561 B = 0.619 *p* = 0.001
Screaming	*R* = 0.492 B = 0.186 *p* = 0.005
Sighing and/or whining	*R* = 0.387 B = 0.119 *p* = 0.031
Moaning and/or groaning	*R* = 0.540 B = 0.196 *p* = 0.002
C (overall score)	*R* = 0.414 B = 0.302 *p* = 0.021
Sleep disturbances	*R* = 0.362 B = 0.153 *p* = 0.045

Abbreviations: *ABQ* Associated Behaviors Questionnaire, *GI* Gastrointestinal, *VB* Verbal behaviors, *MB* Motor behaviors, *C* Changes in overall state, *R* Regression, *B* Beta regression coefficient

Thirty-nine participants (46%) were verbal and 46 (54%) were non-verbal. Among the 39 verbal subjects, 10 children (26%) were in the GI group, whereas among the 46 non-verbal subjects, 20 children (44%) were in the GI group. No statistically significant differences were found between verbal and non-verbal groups as far as the prevalence of GI subjects (*p* = 0.086), the 6GI scores and the Total GSI scores (Table 4).

**Table 4:** Significant differences in ABQ scores between ASD children with and without GI symptoms, and between verbal and non-verbal subjects

	GI children *n* = 30	No-GI children *n* = 55	*p* ≤ 0.01* ≤ 0.001**	Verbal children *n* = 39	Non-verbal children *n* = 46	*p* ≤ 0.01* ≤ 0.001**
6-GI Scores	4.43 ± 1.28	0.82 ± 0.80	< 0.001**	1.74 ± 1.74	2.39 ± 2.16	NS
Total GSI Scores	6.73 ± 1.70	2.04 ± 1.53	< 0.001**	3.10 ± 2.38	4.20 ± 2.97	NS
Associated Behaviors						
Total Score	14.21 ± 5.75	9.33 ± 6.03	0.001**			
VB (overall score)	3.67 ± 2.29	2.55 ± 1.87	0.017			
MB (overall score)	6.93 ± 4.31	4.87 ± 3.63	0.022			
C (overall score)	2.67 ± 1.60	1.91 ± 1.59	0.039			
Frequent clearing of throat, swallowing and/or tics				0.16 ± 0.44	0.53 ± 0.63	0.003
Sighing and/or whining	0.89 ± 0.63	0.53 ± 0.60	0.012			
Moaning and/or groaning	0.78 ± 0.70	0.34 ± 0.52	0.002*	0.34 ± 0.48	0.62 ± 0.70	0.044
Facial grimacing	0.78 ± 0.58	0.31 ± 0.47	< 0.001**			
Constant eating/drinking/swallowing	0.80 ± 0.82	0.22 ± 0.50	< 0.001**			
Mouthing behaviors as chewing on clothes, pica				0.29 ± 0.56	0.64 ± 0.75	0.022
Application of pressure to abdomen	0.61 ± 0.79	0.25 ± 0.44	0.010*			
Tapping behaviors	0.15 ± 0.36	0.02 ± 0.13	0.021			
Sleep disturbances	1.21 ± 0.69	0.64 ± 0.78	0.001**			

Abbreviations: *GI* Gastrointestinal, *No-GI* Non-Gastrointestinal, *GSI* Gastrointestinal Severity Index, *VB* Verbal behaviors, *MB* Motor behaviors, *C* Changes in overall state

We found significantly higher scores for the GI group on the “ABQ Total Score”, and in all the three ABQ cluster scores (Table 4). Furthermore, statistically significant higher mean scores were detected in the GI group than in the No-GI group in the following items: “sighing and/or whining”, “moaning and/or groaning”, “facial grimacing”, “constant eating/drinking/swallowing”, “application pressure to abdomen”, “tapping behaviors”, and “sleep disturbances”.

Three items of the ABQ were statistically significant higher in the non-verbal group than in the verbal group: “frequent clearing of throat, swallowing and/or tics”, “moaning and/or groaning”, “mouthing behaviors”.

Then, we examined the correlations between the 6-GI symptoms and the ABQ Total Score in the verbal group (R = 0.310, B = 1.246, *p* = 0.090) and in the non-verbal group (R = 0.481, B = 1.634. *p* = 0.006).

In the verbal group, the 6-GI Score was positively correlated with “frequent clearing of throat, swallowing and/or tics” (*p* = 0.022), “direct verbalization about pain or stomach” (*p* = 0.018), “constant eating/drinking/swallowing” (*p* = 0.007), and “sleep disturbances” (*p* = 0.014).

The statistically significant correlations between the 6-GI score and the 3 summary clusters (VB, MB and C) and with each AB of the verbal subjects are reported in Table 3.

In the non-verbal group, the 6-GI score was positively correlated with VB total score (*p* = 0.001), “screaming” (*p* = 0.005), “sighing and/or whining” (*p* = 0.031), “moaning and/or groaning” (*p* = 0.002), C total score (*p* = 0.021), and with “sleep disturbances” (*p* = 0.045).

The statistically significant correlations between the 6-GI score and the three summary clusters (VB, MB and C) as well as with each AB of the non-verbal subjects are reported in Table 3.

Finally, considering the possible influence of having more internalizing or more externalizing problems on the subject’s expression of abdominal discomfort, we analyzed the correlations between the CBCL scores (Internalizing, Externalizing, Total Problems) and the 6-GI scores. The Internalizing Problems and the Total Problems were significantly correlated with the 6-GI score (*p* = 0.006 R = 0.30, and *p* = 0.036 R = 0.23, respectively). We did not find significant correlation between the 6-GI scores and the Externalizing Problems.

## Discussion

Over the past years, the evaluation and the understanding of GI symptoms in ASD individuals are considerably improved, and crucially the importance of considering behavioral symptoms as a possible expression of GI distress is repeatedly emerging [13, 15, 19, 22, 28, 29].

To the best of our knowledge, this is the first study that seeks to investigate the possible association between all the AB and GI symptoms, with the aim of contributing to a better comprehension of the way by which ASD subjects signal the presence of a GI problem.

Results pointed out to significantly higher scores in ABQ Total scores as well as in the scores of the three clusters proposed by Buie and colleagues [24] in subjects with significant GI symptoms than in subjects without, with some AB significantly associated to the presence of an underlying GI problem. The abovementioned association was maintained independently from clinical variables -involving the adaptive functioning and emotional-behavioural psychopathology- that distinguish GI from No-GI subjects. These findings could provide a new interpretation for some events that are usually considered part of the autistic symptomatology [15, 45]. The sudden appearance of self- and other-directed aggression, an increase in motor activity or in repetitive behaviors and a worsening of global adaptive behaviors in ASD subjects could suggest clinicians starting or increasing a psychopharmacological treatment that instead could be prevented through a correct diagnosis and appropriate treatment of the underlying GI problem. Accordingly, the contribution of GI symptoms to the onset of sudden irritability or aggressive behavior in non-verbal ASD children has already been reported [28, 46]. In addition, we confirm our previous findings showing that ASD subjects with GI problems have a worse clinical functioning than ASD subjects without GI problems, independently from the severity of autistic symptoms [14]. Of note, the presence of more substantial internalizing or externalizing symptoms may influence the subject’s expression of abdominal discomfort.

Behaviors like sighing and/or whining, moaning and/or groaning, facial grimacing and sleep disturbances are easily associable to the discomfort of an underlying GI problem. Other manifestations like constant eating/drinking/swallowing, application pressure to abdomen and tapping behaviors are more difficult to predict in these subjects. Since the action of constantly eating small amounts of food or repetitively swallowing with a feeling of obstruction (dysphagia) are key symptoms of gastro-esophageal reflux (GER), we can hypothesize a link between “constant eating/drinking/swallowing” behavior and GER. This result could be particularly relevant, since GER is a problem difficult to identify in the early stage, which often leads to medical complications, and usually requires invasive diagnostic techniques [47]. “Holding the abdomen” has already been referred to constipation in children and adolescents with ASD [48], and it could be the only visible sign of a health issue with a high rate of hospitalization [49]. It has also been associated with GERD, intestinal inflammation, malabsorption and maldigestion [24, 29]. “Tapping behaviors” have been only recently considered as a possible expression of GI problems [24], since they were frequently interpreted as a behavior specific to autism [50]. In all these cases, the modalities of onset (e.g. sudden or gradual) and other anamnestic information are needed for symptom characterization and proper treatment options.

Previously, it has been suggested that the presence of these AB is not useful per se for the identification of GI problems, since they are very common also in children without GI problems [28]. Buie and colleagues [24] pointed out that the motor behaviors are not specific to GI problems, but may be indicative of pain or discomfort arising also in other parts of the body. Our study found a positive correlation between the 6-GI score and all the three ABQ clusters, showing for the first time a specificity of these behaviors. In particular, some specific behaviors seem to represent a direct expression of a GI problem in ASD subjects: vocal behaviors like “frequent clearing of throat, swallowing and/or tics”, “screaming”, “sighing and/or whining”, “moaning and/or groaning”, “direct verbalization about pain or stomach”, and motor behaviors like “facial grimacing”, “constant eating/drinking/swallowing”, “application of pressure to abdomen” and “aggressive behaviors”, as well as “changes in overall states”.

As far as the comparison between verbal and non-verbal children, the two groups showed several significant differences when the 6-GI Score was taken into account. “Mouthing behaviors” seemed to differentiate verbal from non-verbal subjects. “Frequent clearing of throat, swallowing and/or tics”, “direct verbalization about pain or stomach” and “constant eating/drinking/swallowing” are symptoms that characterized the verbal group with higher 6-GI scores, whereas “screaming”, “sighing and/or whining” and “moaning and/or groaning” were more representative for the non-verbal group with higher 6-GI scores. Notably, “sleep disturbances” were present in both groups, in agreement with previous literature [28, 51, 52]. Therefore, non-verbal subjects with a more relevant GI symptomatology show more frequently vocal behavior and changes in the global state than verbal subjects with a GI disturb. Interestingly, there were no significant differences in the prevalence and severity of GI symptoms between verbal and non-verbal subjects. In a recent review, the authors concluded that it was not possible to examine the association between GI symptoms and verbal skills in subjects with ASD, since the majority of studies did not distinguish the prevalence of GI symptoms on the basis of expressive language level (e. g. verbal vs non-verbal subjects). By evaluating the inverse relationship, previous investigations on relatively small samples detected both lower verbal abilities [19] and no differences of verbal skills [6, 53] in ASD children with GI problems compared to ASD children without GI problems. This result supports the view that non-verbal ASD children differ from verbal ASD patients only in the modalities of GI discomfort expression, but not in the prevalence or global intensity of GI symptoms. We can hypothesize that non-verbal children activate a minimally verbal behavior to report a physical discomfort (AB as “screaming”, “sighing and/or whining” and “moaning and/or groaning”), while verbal subjects more frequently verbalize their pain (AB as “direct verbalization about pain or stomach”). Importantly, the relatively low average age and the high proportion of subjects not-evaluable at the psychometric test (i.e. more functionally compromised) could partly explain the significantly higher presence of behaviors like “frequent clearing of throat, swallowing and/or tics” in verbal subjects, as well as the low prevalence of “delayed echolalia that includes reference to pain or stomach” in both groups and in the total sample. Crucially, this result indicates that verbal subjects with autism use direct verbalizations, rather than echolalia, to signal a discomfort.

It is important to be aware of the atypical presentation of GI symptoms [54]. Usually, the questionnaires that investigate the GI symptoms in ASD children are based almost exclusively on the symptoms reported by the parents rather than on the visible signs [6, 20, 52, 53, 55, 56].

The presence of significant GI symptoms has been evaluated through the administration of parent-report questionnaire and this could represent a limitation, particularly in younger subjects or in children with more severe autistic symptoms [15]. However, a high concordance between parental reports and gastroenterologist evaluations has been reported [19, 29].

In the future, the sensitivity and the specificity of our results could be enhanced by providing a complete gastroenterological evaluation in ASD subjects with higher GI total scores or at least in the positive cases [29]. Conversely, our protocol could be recommended in hospitals with limited economic resources.

The GSI does not evaluate the presence of some frequently reported GI symptoms, such as GER and vomiting and the cut-off we used was based on the examination of the previous literature, but it is not yet validated. Furthermore, a potential bias derived from the expected numerical disparity between GI and No-GI subjects may affect interpretation of results. Finally, our results are limited to functional GI disorders: therefore, additional research on the associated behaviors in children with organic GI problems is needed.

In addition, a future comparison between these results and those obtained from a group of age and sex matched typical controls could be useful in order to better understand the features of the AB related to the GI symptomatology, independently from an ASD diagnosis.

## Conclusions

In summary, our results encourage researchers to adapt the current tools used to investigate GI symptomatology in ASD subjects, through the addition of items that are based also on objective measures. The evaluation of AB may be useful to identify the presence of GI problems in the ASD populations, and especially in non-verbal ASD children. Moreover, it is important to enhance clinicians’ awareness of the high co-occurrence of GI symptoms in ASD subjects, so that targeted investigations can be undertaken [45].

Our understanding of GI problems in ASD in relation with AB has come a long way, but further studies and more systematic research are warranted.

## Data Availability

The datasets generated and/or analyzed during the current study are not publicly available due the privacy policy (containing information that could compromise research participant privacy/consent), but are available from the corresponding author on reasonable request and with permission of parents of the involved children.

## Abbreviations

AB: Associated Behaviors
ABQ: Associated Behaviors Questionnaire
ADOS-2: Autism Diagnostic Observation Schedule-Second Edition
ANOVA: Analysis of Variance
ASD: Autism Spectrum Disorder
C: Changes in Overall State
CBCL: Child Behavior Checklist
DNA: Desoxyribonucleic Acid
DSM-5: Diagnostic and Statistical Manual of Mental Disorders-5th Edition
F: Females
FRA-X: Fragile X
GER: Gastro-esophageal reflux
GERD: Gastro Esophageal Reflux Disease
GI: Gastrointestinal
GMDS-ER: Griffiths Mental Development Scales-Extended Revised
GSI: Gastrointestinal Severity Index
IBD: Irritable Bowel Disease
M: Males
MB: Motor Behaviors
RBS-R: Repetitive Behaviors Scale Revised
VABS: Vineland Adaptive Behavior Scales
VB: Verbal Behaviors
